# An Aggressive Presentation of Anti-melanoma Differentiation-Associated Gene 5 (MDA-5)-Positive Dermatomyositis With Rapidly Progressive Interstitial Lung Disease: A Case Report

**DOI:** 10.7759/cureus.111003

**Published:** 2026-06-16

**Authors:** Zamanali Khakhar, Shreedhi M Patel, Rajiv Patel, Soraiya Manji, Fredrick Otieno, Sayed K Ali

**Affiliations:** 1 College of Medicine, University of Nairobi, Nairobi, KEN; 2 Department of Internal Medicine, Aga Khan University Hospital, Nairobi, KEN

**Keywords:** anti-mda-5 amyopathic dermatomyositis, anti-mda-5 antibodies, dermatomyositis, dermatomyositis-associated interstitial lung disease (dm-ild), rapidly progressive interstitial lung disease

## Abstract

Anti-melanoma differentiation-associated gene 5 (MDA-5) dermatomyositis is an uncommon condition that is frequently complicated by rapidly progressive interstitial lung disease (RP-ILD), resulting in a poor prognosis. Due to its aggressive course, management is often challenging and requires timely interventions to improve outcomes. This case of a female patient with anti-MDA-5-positive DM and RP-ILD aims to document this unique and uncommon condition, its aggressive clinical course, associated complications, diagnostic challenges, and management considerations.

## Introduction

Dermatomyositis (DM) is a rare immune-mediated inflammatory myopathy, commonly characterized by distinctive cutaneous manifestations and subacute, progressive, symmetrical proximal muscle weakness [1]. It is a subgroup of idiopathic inflammatory myopathies (IIM). Other established subgroups of IIM include overlap myositis, inclusion body myositis, anti-synthetase syndrome, and immune-mediated necrotizing myopathy, all of which are heterogeneous in their clinical and pathological presentations [2]. It is rarely encountered, with an estimated incidence of less than 10 cases per million people annually. It commonly affects individuals between the ages of 40 and 50 years and has a higher female predominance [3].

Anti-melanoma differentiation-associated gene 5 (MDA-5) DM is a rare subtype of DM and presents as clinically amyopathic dermatomyositis (CADM). It is associated with distinct clinical phenotypes, rapidly progressive interstitial lung disease (RP-ILD), and generally carries a poor prognosis [4,5].

This case of a female patient with anti-MDA-5-positive DM and RP-ILD aims to document this unique and uncommon condition, its aggressive clinical course, associated complications, diagnostic challenges, and management considerations. It further aims to contribute to the limited number of existing cases reporting anti-MDA-5 DM with rapidly progressive pulmonary involvement.

## Case presentation

A 33-year-old female of African descent presented to our rheumatology service in May 2025 with a three-month history of a progressive itchy rash involving her anterior chest wall, earlobes, forearms, and dorsal hands. She also had episodic polyarthralgia with hand numbness, facial puffiness in the morning, and recurrent nasal ulcers during this period. Prior to the onset of these symptoms, she had a hospital admission for a febrile illness that was treated with antibiotics. Examination was significant for an extensive, slightly raised macular rash involving her anterior chest wall extending to the neck in an almost V-shaped pattern, earlobes, distal forearms, and knuckles bilaterally. The remainder of the systemic examination was largely unremarkable. An extensive laboratory evaluation was performed; myositis antibodies, including anti-MDA-5 and anti-PL-7 antibodies, were positive. Based on the above, a diagnosis of amyopathic DM was established, and the patient was commenced on prednisone (tapering dose) and mycophenolate mofetil. She was seen one month later and had marked resolution of the cutaneous lesions, with no additional symptoms reported.

The patient was unfortunately lost to follow-up until January 2026, when she presented to the emergency department with a one-month history of progressive malaise, limb weakness with difficulty ambulating and raising both arms, and episodes of lightheadedness. She also had a productive cough, progressive dyspnea, and chest pain. Her relatives noted episodes of fever, progressive hair thinning, and unintentional weight loss (from 62 kg to 42 kg over a period of one month).

On examination, the patient appeared ill, wasted, and dehydrated. She had marked jaundice, severe pallor, and a puffy face. She was tachypneic and tachycardic. Loss of muscle bulk and joint stiffness were also identified. Respiratory examination revealed bilateral crackles in the lung fields. Epigastric tenderness was elicited on abdominal palpation. Dermatologic findings included a generalized hyperpigmented rash. Diffuse hair thinning was also present.

**Figure 1:**
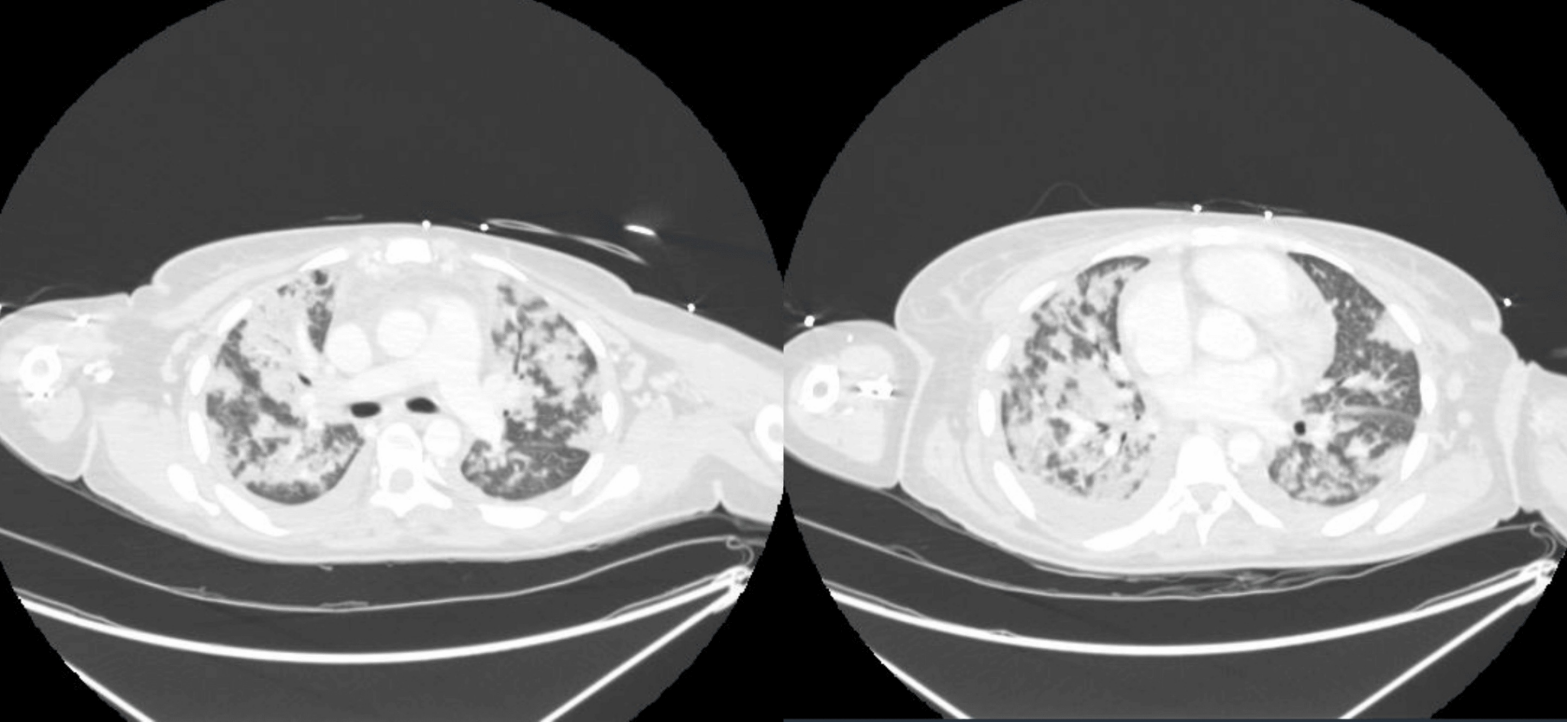
Bilateral, patchy, diffuse fibrotic changes of the lung parenchyma consistent with interstitial lung disease.

CT of the chest revealed bilateral, patchy, diffuse fibrotic changes of the lung parenchyma. The findings were consistent with interstitial lung disease (ILD) (Figure 1).

CT imaging demonstrated mild hepatomegaly measuring 17 cm, without evidence of intrahepatic or extrahepatic biliary duct dilatation. Mild splenomegaly was also noted, with multiple scattered hypodense splenic lesions, the largest measuring 0.8 cm. Prominent splenic hilar lymph nodes with necrotic centers were also present.

Bronchoalveolar lavage was negative for malignancy. Culture grew *Candida albicans*, for which the patient was treated with caspofungin 50 mg intravenously daily. The respiratory viral panel was negative for viral pathogens, including influenza, COVID-19, and *Pneumocystis jirovecii* pneumonia (PJP). Sputum culture for tuberculosis (TB) was negative at six weeks. Urinary TB lipoarabinomannan (LAM) was also negative.

Laboratory investigations revealed significant anemia and electrolyte derangements, such as hyponatremia, that improved to normal values the following day with adequate hydration. She had no bleeding tendencies. Iron studies showed elevated ferritin and transferrin saturation, suggesting that the anemia was due to chronic inflammation.

Liver function tests were elevated, with increased aspartate aminotransferase (AST), gamma-glutamyl transferase (GGT), alkaline phosphatase (ALP), and bilirubin levels, along with hypoalbuminemia, all potentially attributable to the underlying autoimmune disease (Table 1). Prior serological testing was positive for anti-MDA-5 and anti-PL-7 antibodies. Serologic testing for hepatitis B surface antigen (HBsAg) and hepatitis C virus (HCV) antibodies was negative. Human immunodeficiency virus (HIV) testing was also negative. Autoimmune hepatitis was excluded, given the absence of corroborative serological markers.

**Table 1:** Patient laboratory results. BUN, blood urea nitrogen; LDH, lactate dehydrogenase; INR, international normalized ratio; GGT, gamma-glutamyl transferase; AST, aspartate aminotransferase; ALT, alanine aminotransferase

Test	Results	Reference
White blood count	9.02 × 10^9^/L	4.00 - 10.00 × 10^9^/L
Hemoglobin	4.00 g/dL	13.00 - 17.00 g/dL
Hematocrit	13.50%	40.00 - 50.00%
Platelets	156 × 109/L	150 - 400 × 109/L
Sodium	130.60 mmol/L	136.00 - 145.00 mmol/L
Potassium	4.61 mmol/L	3.50 - 5.10 mmol/L
Chloride	101.20 mmol/L	98.00 - 107.00 mmol/L
Bicarbonate	24.30 mmol/L	22.00 - 29.00 mmol/L
BUN	4.48 mmol/L	3.20 - 8.20 mmol/L
Creatinine	50.20 µmol/L	62.00 - 115.00 µmol/L
LDH	1286	120 - 246 U/L
Ferritin	4875	13 - 150 ng/mL
INR	1.04	0.8 to 1.1
Total bilirubin	47.5	(5.0 - 21.0) µmol/L
Direct bilirubin	35.5	(0.0 - 5.0) µmol/L
GGT	524.00	(0.00 - 38.00) U/L
AST	126.00	(0.00 - 34.00) U/L
ALT	30.70	(0.00 - 49.00) U/L
Alkaline phosphatase	726.00	(46.00 - 116.00) U/L
Albumin	25.40	(32.00 - 48.00) g/L

A multidisciplinary approach was required, involving rheumatology, pulmonology, internal medicine, and intensive care. The patient remained in the intensive care unit during this period. During hospitalization, she developed rapidly progressive respiratory compromise, necessitating early endotracheal intubation. In addition to broad-spectrum antibiotics, she was initiated on high-dose intravenous methylprednisolone (1 mg/kg) for three days, followed by a gradual taper. Cyclophosphamide (500 mg) was then initiated with close monitoring of liver enzymes. Additionally, plasmapheresis was performed as part of her treatment regimen; however, this did not result in clinical improvement.

Despite aggressive interventions, her clinical condition progressively worsened in the intensive care setting. This was primarily due to deteriorating pulmonary function in the context of RP-ILD. The patient ultimately succumbed to her illness.

## Discussion

Anti-MDA-5 antibody-positive DM is a rare immune-mediated disease affecting multiple systems, first documented in Japan [6,7]. It is characterized by inflammation of the skin, lungs, and vasculature and is frequently complicated by RP-ILD, often associated with poor outcomes. Notably, muscle involvement in this subtype of DM is minimal compared to other phenotypes of DM [8]. An extensive literature review was conducted, and to the best of our knowledge, no previously reported cases of anti-MDA-5-positive DM have been documented in our setting or within the wider sub-Saharan African region, highlighting the rarity of this condition in our context and the associated challenges in its diagnosis and management. It remains a rare disease representing approximately 2% of IIM, with an overall prevalence of 7-60%. Higher prevalence has been reported in Asian populations, and it has a female predominance, with a female-to-male ratio ranging from 0.6 to 7.3 [9].

MDA-5 is a protein found in the cytosol and is essential for host antiviral immune responses. It detects viral RNA and, upon activation, triggers the production of type I interferons (IFN-I) and pro-inflammatory cytokines [9].

DM is a complex disease, and its pathogenesis remains largely unknown. However, various hypothesized mechanisms are thought to underlie the pathological process, most notably involving a complex interaction between genetic and environmental factors [8].

Firstly, an inherent genetic predisposition exists in individuals carrying human leukocyte antigen (HLA) genotypes, namely HLA-DRB10101 and HLA-DRB10405. These alleles present anti-MDA-5-derived peptides to T lymphocytes, driving the generation of autoantibodies against native proteins [8].

Aberrant concurrent activation of both the innate and adaptive arms of the immune system underlies the immunopathogenesis of anti-MDA-5 DM. After exposure to an initial trigger, dendritic cells chronically produce pro-inflammatory mediators such as IFN-I. This process has been termed an interferonopathy. Macrophages play a pivotal role in propagating this process; activated macrophages directly cause fibrosis and are responsible for the cutaneous and pulmonary manifestations of the disease. Further activation of the complement system ensues, resulting in the generation of the membrane attack complex (MAC), which causes direct injury to endothelial cells. Downstream activation of humoral immunity perpetuates this inflammatory process through the production of B cells that contribute to autoantibody production. Numerous cytokines, including interleukin (IL)-6, IL-10, IL-18, macrophage colony-stimulating factor, and IFN-α, have been implicated in the pathogenesis of DM [1,10,11].

The above mechanisms are thought to be triggered by viral infections such as HSV-1 and Enterovirus B. Recognition of viral RNA results in MDA-5-initiated formation of IFN-I and immune activation, leading to downstream effects of immune-mediated self-injury [8]. Notably, recent research has also implicated COVID-19 infection in the pathogenesis of anti-MDA-5 DM. SARS-CoV-2 and anti-MDA-5 DM share a common pathogenic axis centered on a strong IFN-I immune pathway. Both cause endothelial damage and subsequent platelet activation, resulting in thrombosis [12].

Early and precise recognition of pathognomonic findings is key to the early diagnosis of anti-MDA-5 DM. Primary clinical signs include cutaneous manifestations in the form of Gottron’s papules and heliotrope rash [10]. Regrettably, atypical presentations of the disease are often misdiagnosed as rheumatoid arthritis, psoriasiform dermatitis, or inflammatory arthritis, ultimately leading to delayed specialized care [13].

The management of DM with ILD can be simplified into two-drug therapy, high-dose corticosteroids (such as prednisone) and a calcineurin inhibitor, and triple therapy, high-dose corticosteroids, calcineurin inhibitors, and cyclophosphamide. Nonresponsive cases that require additional therapies include plasma exchange, rituximab, and the JAK inhibitor tofacitinib. There is little research guiding treatment intensification, including criteria for plasma exchange, timing of initiation, and the number and frequency of sessions, especially for patients presenting with ILD or RP-ILD who are already critically ill at the time of diagnosis [10].

Moreover, combined immunosuppressive therapy is associated with undesirable side effects, including increased susceptibility to opportunistic infections, reactivation of latent disease, and drug-mediated kidney injury [10]. In the case reported by Risal et al., the authors illustrate the profound difficulties in managing anti-MDA-5 DM within resource-constrained environments. The study highlights various systemic hindrances in the diagnosis and management of anti-MDA-5 DM. The authors describe a scarcity of rheumatologists as a major drawback, particularly in rural areas, which further restricts patient access to healthcare. Diagnostic capabilities are further complicated by the lack of localized testing capacities, which warrant out-of-country sourcing that has both time and financial constraints [13].

Anti-MDA-5 DM presents with distinct disease-specific features in addition to those that overlap with classical DM. It is characterized by unique cutaneous manifestations as well as pathognomonic skin manifestations of DM, such as periorbital heliotrope rash, erythematous rash in a shawl distribution, and Gottron’s papules. Muscle involvement may be minimal or absent, with recent evidence indicating that patients with anti-MDA-5 DM frequently exhibit little to no muscle disease. Pulmonary involvement is often rapidly progressive, which distinguishes it from other forms of DM. RP-ILD involves inflammation and resultant fibrosis and carries a poor prognosis in anti-MDA-5 DM. Additionally, anti-MDA-5 DM presents with unique cutaneous lesions that include palmar papules and skin ulcerations that manifest as deep, painful ulcers localized over Gottron’s papules [9,14].

Symmetric inflammatory arthropathy and arthralgias are common manifestations of anti-MDA-5 DM and are commonly associated with hand swelling, which often overlaps clinically with rheumatoid arthritis [14]. Hall et al. highlighted several cases of anti-MDA-5 antibody-positive patients who presented with phenotypes similar to those of antisynthetase syndrome, with at least three clinical features, including fever, non-erosive arthritis, Raynaud’s phenomenon, mechanic’s hands, and ILD [15].

RP-ILD develops in approximately half of the patients with anti-MDA-5 DM and represents a major determinant of prognosis. It is often associated with a poor outcome, with mortality rates approaching 50% despite early and aggressive interventions, predominantly in the early stages of the disease [9,16]. Upon detection of anti-MDA-5 antibodies, a high risk of RP-ILD should be recognized, prompting early evaluation with appropriate investigations, including high-resolution computed tomography (HRCT) and pulmonary function tests, to assess the extent of pulmonary involvement and associated functional impairment [4]. Given the aggressive and rapidly progressive nature of the disease in patients with anti-MDA-5 DM, early identification of individuals at high risk for disease progression and increased mortality is important to initiate timely and appropriate therapeutic interventions. A pooled analysis of 1153 patients across 15 studies was conducted by Yang et al., who identified several risk factors associated with increased mortality in anti-MDA-5 DM-ILD, including advanced age, smoking, fever, elevated C-reactive protein (CRP), RP-ILD, leukocytosis, elevated serum KL-6 levels, hyperferritinemia (ferritin >800 ng/mL), and lymphopenia (lymphocyte count <1.1 × 10⁹/L) [16]. Evidence from multiple studies has shown that the anti-MDA-5 antibody titer in serum was significantly correlated with disease activity and death [7].

Elevated hepatobiliary enzymes, as seen in this case, have been reported to correlate with ILD in anti-MDA-5 DM. One proposed explanation is that anti-MDA-5 antibodies may contribute to alveolar macrophage activation, which leads to inflammatory injury involving multiple organs, including the lungs, skin, and liver [17].

Initial evaluation includes measurement of muscle enzymes in suspected cases of DM based on clinical findings and history. These include creatine kinase, lactate dehydrogenase (LDH), AST, alanine aminotransferase (ALT), and aldolase [1]. These are neither entirely specific nor sensitive for DM. The detection of specific autoantibodies can aid in defining the clinical subtypes and clinical spectrum of DM. Myositis-specific autoantibodies (MSAs) are present in approximately 30% of patients with DM. The most common among the MSAs are anti-synthetase antibodies. Notably, the presence of these autoantibodies is linked to distinct clinical manifestations and potential complications that influence prognosis and therapeutic decision-making [1,15].

Electromyography is used to identify the most affected muscle groups for further evaluation using a muscle biopsy. However, in patients with anti-MDA-5 DM, muscle biopsies are not significant, as they most often appear normal. In the presence of characteristic skin manifestations, a skin biopsy can be performed. In patients with anti-MDA-5 DM, histology of skin biopsies reveals vacuolar changes, apoptotic keratinocytes, and CD8+ lymphocyte infiltration at the dermoepidermal junction, a feature known as interface dermatitis, which is also shared by systemic lupus erythematosus and lichen planus [1,9]. An ideal approach to diagnostics should therefore include clinical assessment, laboratory investigation, and tissue biopsies.

Delayed follow-up in our patient and prolonged interruption in treatment led to profound deterioration of lung function secondary to RP-ILD, which was diagnosed following exclusion of multiple alternative respiratory conditions. This delay in care likely contributed to advanced disease progression, rendering subsequent management challenging and aggressive. Despite treatment with high-dose intravenous methylprednisolone followed by a gradual taper and subsequent cyclophosphamide therapy, there was no significant clinical improvement.

These outcomes contrast with findings from previously reported cases, where early initiation of aggressive immunomodulatory therapy was associated with attenuation of disease progression [4]. Our case highlights the importance of early diagnosis, prompt initiation of treatment, adherence to therapy, and regular follow-up. In patients with DM who test positive for anti-MDA-5 antibodies, there should be increased awareness of potential complications, which may facilitate earlier interventions, thereby reducing the risk of irreversible respiratory compromise and adverse outcomes.

## Conclusions

Anti-MDA-5 DM is a clinically heterogeneous condition that requires timely assessment and therapeutic intervention due to its rapid progression and pulmonary deterioration. Awareness of this condition, particularly in settings where rheumatologists may not be readily accessible, can expedite referral and management. This case highlights the rapidly progressive nature of this condition and the high risk of mortality associated with it, thereby emphasizing the importance of early identification and appropriate intervention.
